# Supplementary material for: Investigation of Tannic Acid Crosslinked PVA/PEI-Based Hydrogels as Potential Wound Dressings with Self-Healing and High Antibacterial Properties
Source: Gels. 2024 Oct 23;10(11):682. doi: 10.3390/gels10110682 (PMC11593458; doi:10.3390/gels10110682)
Supplement: Supplementary file 1 [file gels-10-00682-s001.zip › gels-3225004-supplementary.pdf]

Supplementary Materials

Investigation of Tannic Acid Cross-Linked PVA/PEI-Based Hydrogels as Potential Wound Dressings with Self-Healing and High Antibacterial Properties

Nimet Rumeysa KARAKUŞ[1,2], Serbülent TÜRK[2,3], Gamze GUNEY ESKILER[4], Marat SYZDYKBAYEV[5], Nurbol O. Appazov[5,6,*] and Mahmut ÖZACAR[2,7,*]

1 Sakarya University, Institute of Natural Sciences, Department of Biomedical Engineering, 54187, Sakarya, Turkiye; nmtrmys.06@gmail.com

2 Sakarya University, Biomaterials, Energy, Photocatalysis, Enzyme Technology, Nano & Advanced Materials, Additive Manufacturing, Environmental Applications and Sustainability Research & Development Group (BIOENAMS R & D Group), 54050 Sakarya, Turkiye

3 Sakarya University, Biomedical, Magnetic and Semiconductor Materials Application and Research Center (BIMAS-RC), 54187 Sakarya, Turkiye; serbulentturk@sakarya.edu.tr

4 Sakarya University, Faculty of Medicine, Department of Medical Biology, Sakarya, Turkiye; gamzeguney@sakarya.edu.tr

5 Laboratory of Engineering Profile "Physical and Chemical Methods of Analysis", Korkyt Ata Kyzylorda University, Aiteke bi Str., 29A, Kyzylorda 120014, Kazakhstan; marat.1980@mail.ru (M.S.), nurasar.82@korkyt.kz (N.A.)

6 KazEcoChem LLP, D.Konaev Str. 12, Astana, 010010, Kazakhstan; sales@kazecochem.kz

7 Sakarya University, Faculty of Science, Department of Chemistry, 54050, Sakarya, Turkiye

* Correspondence: nurasar.82@korkyt.kz (N.O.A.); mozacar@sakarya.edu.tr (M.O.)

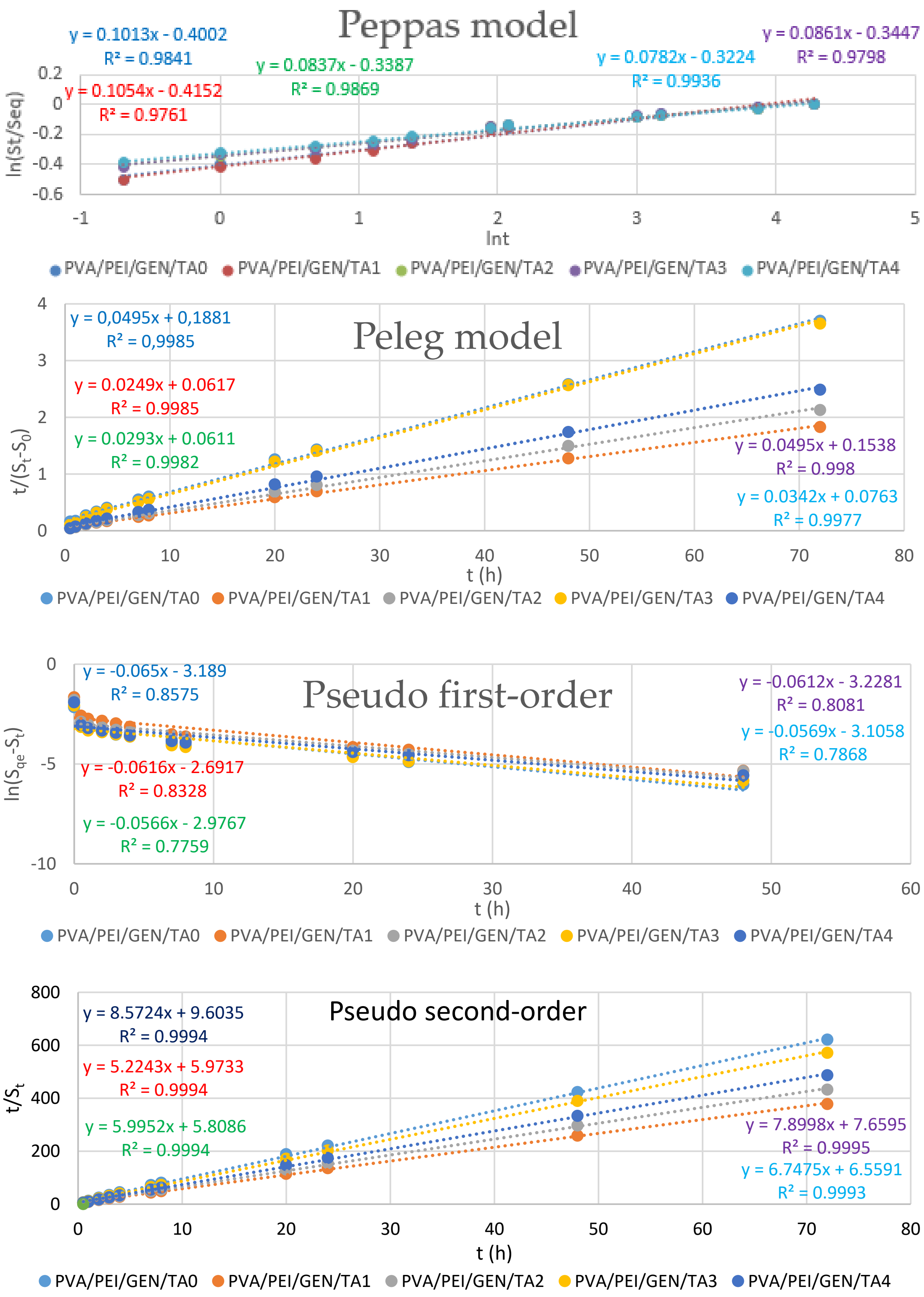

Figure S1. Different kinetic models for swelling of hydrogels.

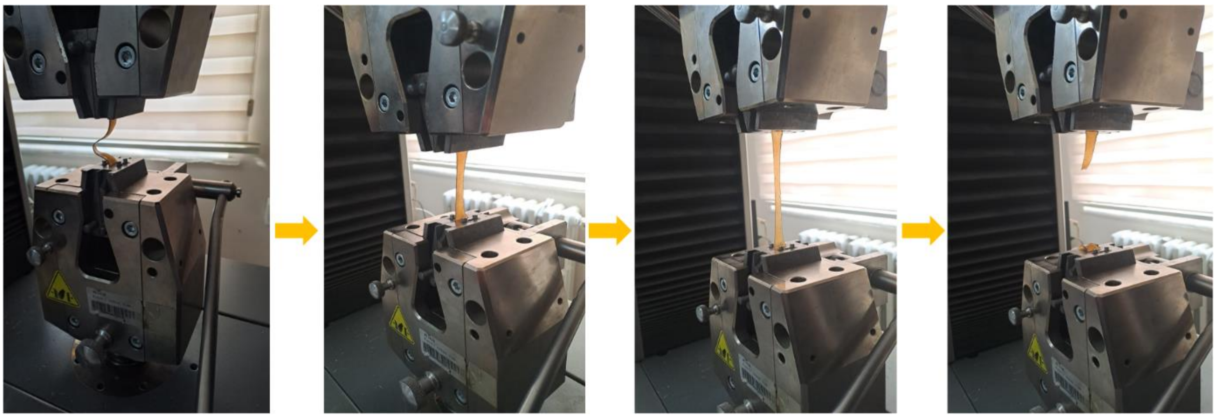

Figure S2. Tensile test applied to PVA/PEI hydrogel samples.

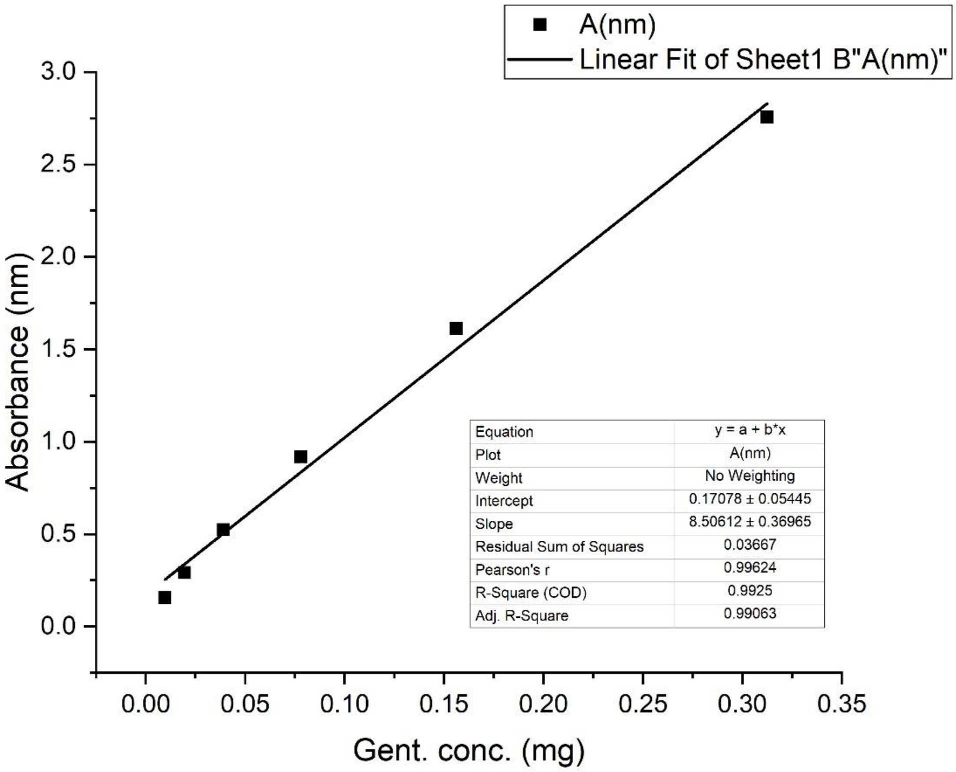

Figure S3. Gentamicin standard chart created for controlled drug release testing.

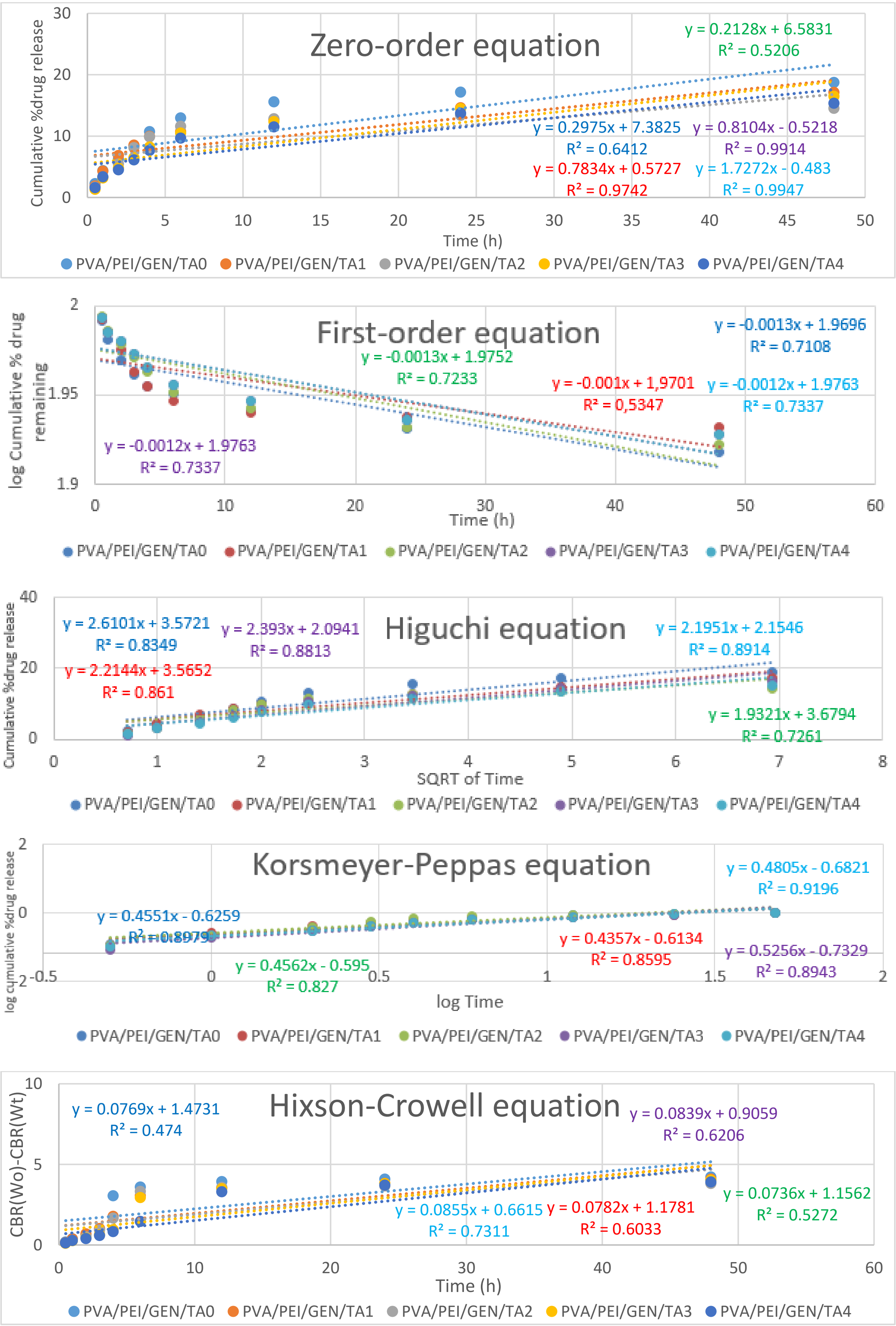

Figure S4. Various kinetic models for GEN release of hydrogels

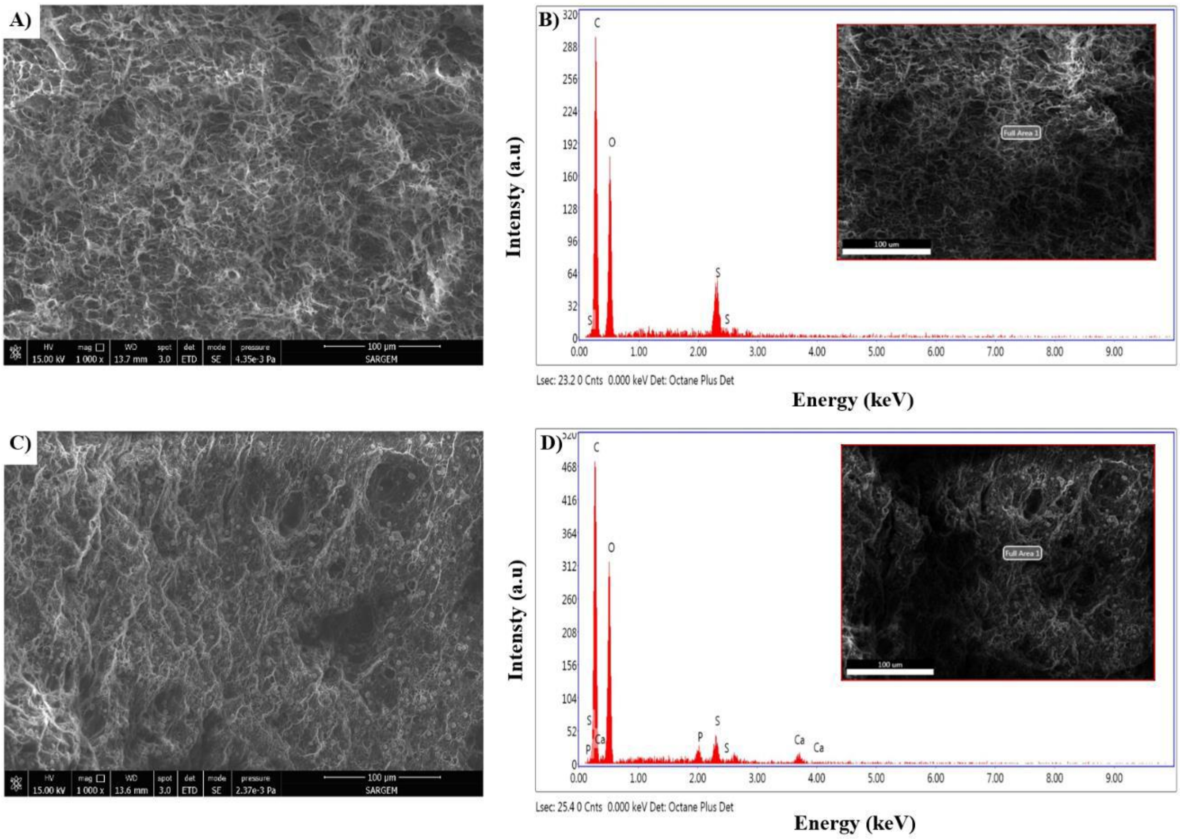

Figure S5. A, B) FESEM image and EDS analysis of PVA/PEI/GEN/TA0 sample, C, D) FESEM image and EDS analysis of PVA/PEI/GEN/TA1.

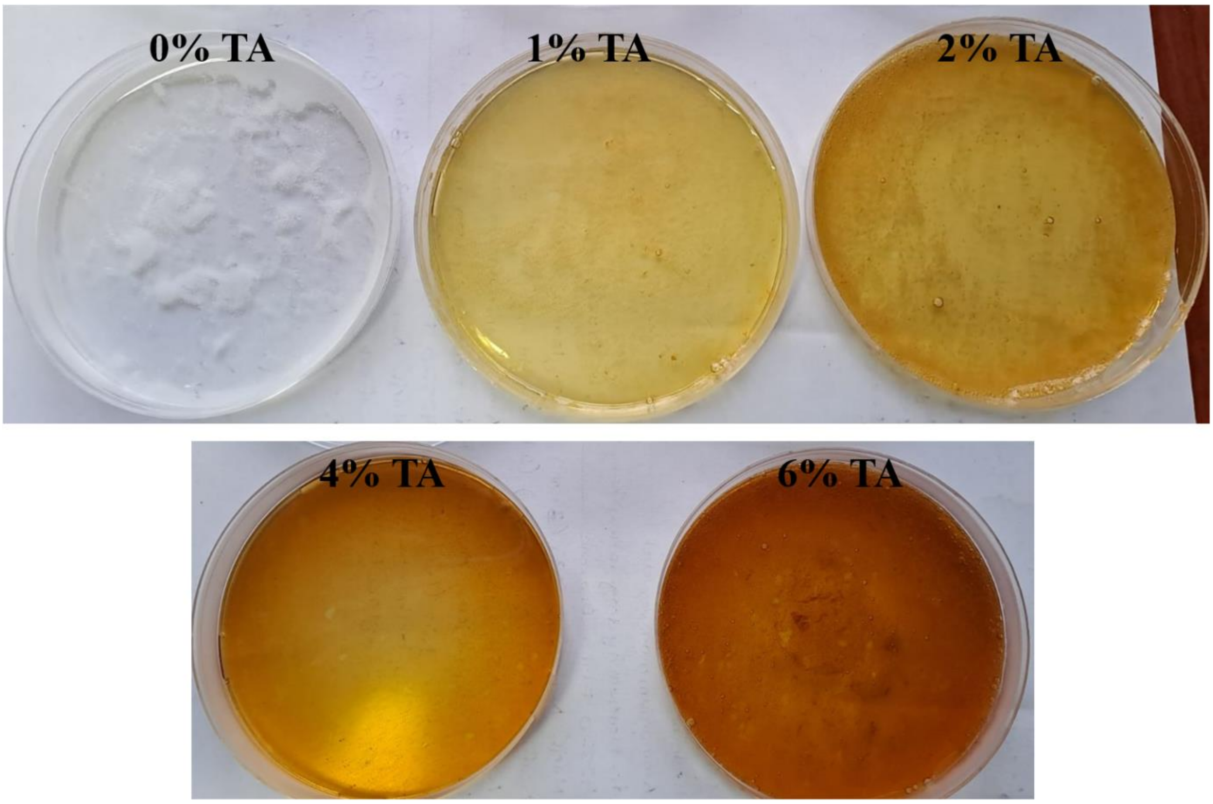

Figure S6. PVA/PEI-based hydrogel samples prepared for characterization tests.

Table S1. Composition and ratios of PVA/PEI based hydrogel samples.

	PVA:PEI (mg:mL)	TA (%)	LiCl (M)	GEN (mg:mL)
PVA/PEI/TA0	5:1	-	1	-
PVA/PEI/TA1	5:1	1	1	-
PVA/PEI/TA2	5:1	2	1	-
PVA/PEI/TA3	5:1	4	1	-
PVA/PEI/TA4	5:1	6	1	-
PVA/PEI/GEN/TA0	5:1	-	1	2
PVA/PEI/GEN/TA1	5:1	1	1	2
PVA/PEI/GEN/TA2	5:1	2	1	2
PVA/PEI/GEN/TA3	5:1	4	1	2
PVA/PEI/GEN/TA4	5:1	6	1	2

Table S2. The required order pertains to the requisite reagent quantities and purities for the preparation of 1000 mL of SBF.

Order	Reagent	Amount	Purity (%)
1	$NaCl$	8.035 g	99.5
2	$NaHCO_3$	0.355 g	99.5
3	KCl	0.225 g	99.5
4	$K_2HPO_4.3H_2O$	0.231 g	99.0
5	$MgCl_2.6H_2O$	0.311 g	98.0
6	1.0 M HCl	39 mL	-
7	$CaCl_2$	0.292 g	95.0
8	Na_2SO_4	0.072 g	99.0
9	Tris	6.118 g	99.0
10	1.0 M HCl	0-5 mL	-
